# Gold conjugated nanobodies in a signal-enhanced lateral flow test strip for rapid detection of SARS-CoV-2 S1 antigen in saliva samples

**DOI:** 10.1038/s41598-023-37347-y

**Published:** 2023-06-30

**Authors:** Sara Maher, Manal Kamel, Zeinab Demerdash, Hanan El Baz, Omar Sayyouh, Amany Saad, Noha Ali, Faten Salah, Shimaa Atta

**Affiliations:** 1 Immunology Department, Theodor Bilharz Research Institute, Giza, Egypt; 2 Infection Control and Clinical Microbiology, Theodor Bilharz Research Institute, Giza, Egypt

**Keywords:** Immunology, Microbiology

## Abstract

Despite the transfer of COVID-19 from the pandemic to control, we are still in a state of uncertainty about long-term success. Therefore, there is a great need for rapid and sensitive diagnostics to sustain the control status. After several optimization trials, we developed lateral flow test (LFT) strips for rapid detection of SARS-CoV-2 spike 1 (S1) antigen in saliva samples. For signal enhancement of our developed strips, we applied dual gold conjugates. Gold-labeled anti-S1 nanobodies (Nbs) were employed as S1 detector conjugate, while gold-labeled angiotensin-converting enzyme 2 (ACE2) was used as S1 capturing conjugate. In a parallel strip design, we used an anti-S1 monoclonal antibody (mAb) as an antigen detector instead of anti-S1 Nbs. Saliva samples were collected from 320 symptomatic subjects (180 RT-PCR confirmed positive cases and 140 confirmed negative cases) and were tested with the developed strips. In early detection for positive samples with cycle threshold (Ct ≤ 30), Nbs-based LFT strips showed higher sensitivity (97.14%) and specificity (98.57%) than mAb-based strips which gave 90.04% sensitivity and 97.86% specificity. Moreover, the limit of detection (LoD) for virus particles was lower for Nbs-based LFT (0.4 × 10^4^ copies/ml) than for the mAb-based test (1.6 × 10^4^ copies/ml). Our results are in favor of the use of dual gold Nbs and ACE2 conjugates in LFT strips. These signal-enhanced strips offer a sensitive diagnostic tool for rapid screening of SARS-CoV-2 S1 antigen in the easily collected saliva samples.

## Introduction

COVID-19 ongoing pandemic, caused by severe acute respiratory syndrome coronavirus 2 (SARS-CoV-2), is one of the most challenging pandemics in history^1^. Controlling the spread of SARS-CoV-2 relies greatly on accurate and early detection of infected cases^2^. After the COVID-19 outbreak, RT-PCR kits were developed and widely applied for the clinical diagnosis of SARS-CoV-2 infection. Despite their high sensitivity and specificity, nucleic acid techniques have their limitations in terms of cost, time efficiency, sophistication, and degree of pollution^3,4^. Moreover, false-negative test results were a major disadvantage^5^. The development of rapid and sensitive point-of-care tests (POCT) was a demand to control the pandemic^6,7^. Antigen tests are one of the most helpful diagnostic tools for population-wide screening approaches^8^. These tests are a direct reflection of the active infection rather than antibodies-based lateral flow tests^9^. Monoclonal antibodies (mAbs) are successfully applied in the development of different antigen-based diagnostic systems, but their complex, large-size structure, affects their tolerability^10^. Nanobodies (Nbs) are recombinant, antigen-specific, single-domain, variable fragments of camelid heavy chain-only antibodies. Unlike conventional antibodies, nanobodies contain a single variable domain (VHH) and two constant domains (CH2 and CH3). Cloned and isolated Nbs possess unique properties that enable them to excel in conventional antibodies^11^. Nbs are highly robust, easy to manufacture, more stable, and have the ability to recognize hidden or uncommon epitopes. Nbs could retain full antigen-binding potential upon isolation and have been efficiently employed in various diagnostic and therapeutic applications^12^.

Nanomaterials provide additional binding sites for detection antibodies, which lead to the enhancement of signal intensity in diagnostic assays^13^. Among colored reporters, gold nanoparticles, with high color intensity, could raise the sensitivity of LFT^14^. Moreover, other features of gold nanoparticles such as cost-effectiveness, ease of production, stability in dried form, and facile conjugation with biomolecules, make them the ideal candidates to use as signal reporters in industrial applications^15^. In our previous work by Kamel et al., 2022, we employed gold-conjugated Nbs in sandwich ELISA for the detection of SARS-CoV-2 S1 antigen in saliva and nasopharyngeal swabs. The results were very interesting by improving the early detection sensitivity of the test by 93.3%^16^.

The entry of SARS-CoV-2 into host cells is mediated by the interaction between the Spike (S) protein subunit, receptor-binding domain (RBD), and human host cell receptors. One major cell surface receptor is angiotensin-converting enzyme 2 (ACE2), which binds with great affinity to SARS-C0V-2^17,18^.

The nasopharyngeal swab is the recommended method for specimen collection from the upper respiratory tract for COVID-19 diagnostic testing. Several drawbacks including, patient discomfort, headache, or severe bleeding from the nose, mainly in thrombocytopenic patients, could limit the use of swabs especially in serial monitoring or mass test programs^19^. On the other hand, using of saliva or sputum represents an easy, fast, and painless sampling collection way, allowing widespread testing to be conducted. Moreover, different studies indicated that the sensitivity and specificity of saliva for the diagnosis of COVID-19 is similar to that of nasopharyngeal swabs^20,21^.

In this study, we employed dual gold conjugates for signal enhancement of LFT strips. Au-Nbs acted as the detector probe, and Au-ACE2 acted as the capture probe, allowing sensitive measurement of the S1 antigen sandwiched between them. These LFT strips provide a sensitive, rapid, non-invasive method for the SARS-CoV-2 spike protein (S1) detection in saliva samples of COVID-19 patients, which is important for the prevention and control of disease spread among the population.

## Materials and methods

### Materials and equipment

Chloroauric acid (HAuCl4), Bovine serum albumin (BSA), sucrose (C12H22O11), and potassium carbonate (K2CO3, ≥ 99.0%), skimmed milk, were all purchased from Sigma Aldrich, MO, USA. Recombinant human coronavirus SARS-CoV-2 spike glycoprotein S1 (ab 288546), Recombinant anti-SARS-CoV-2 antibodies (ab 281311), Recombinant angiotensin-converting enzyme 2 (ACE2) (ab151852), all from Abcam, Cambridge, UK. SARS-CoV recombinant protein (MBS569928), MERS CoV spike S1 (MBS434229) antigen from MyBioSource, California, US, SARS-CoV-2 S1 nanobodies (AssayGenie, Dublin, Irland), Pierce DAB substrate kit (cat. no. 34002, Thermo Fisher USA). A sample pad and absorption pad (cat no CFSP173000), a glass fiber conjugate pad (cat no. GFDX103000), high flow nitrocellulose membrane (NC) (cat no HF09002XSS) were purchased from Merch Millipore (Darmstadt, Germany). UV-Vis-spectrophotometer (Thermofisher-USA). Manual dispenser (Nanomat 4-CAMAG-laborto), PH meter (Jenway 3510, UK), High-speed centrifuge (Eppendorf, 5430R, Germany). Ultrapure water used throughout was generated from a Millipore Milli-Q water purification system (Billerica, MA, USA). Gel documentation system (Gel Doc XR+) (Biorad, USA) & data analysis by “Image lab” software. JEOL JSM5200 Scanning Electron Microscope, Japan.

### Clinical samples

Saliva samples were collected from 320 individuals (males and females with an average age of 35–65 years old) with COVID-19 symptoms (fever, cough, bone aches, diarrhea, headache, sore throat, skin rash, loss of taste or smell, difficulty in breathing, chest pain or pressure) at COVID-19 outpatient clinic of TBRI (June 2021–April 2022), following WHO guidelines. Using a universal viral transport system (UVS), nasopharyngeal swabs were placed in a viral transport medium (3 ml total volume), sealed securely, and sent for RT-PCR testing. Unstimulated saliva samples were self-collected by the patient early in the morning. Patients were asked to wash their mouths with water and then to spit repeatedly into the sterile cups, which were securely closed and preserved rapidly at − 80 °C till use.

### Methods

#### Ethical approval

This study was approved by the Research Ethics Committee (REC) at Theodor Bilharz Research Institute (TBRI) (PT 623, 2021). The human subjects in this study were enrolled according to REC-TBRI’s ethical standards and the 1964 Helsinki Declaration. Written informed consent forms were obtained from all participants.

#### Preparation and characterization of colloidal gold nanoparticles (AuNPs)

AuNPs (40 nm) were prepared successfully by the chemical reduction method. The protocol was adopted from Frens et al.^22^ with slight modifications according to Borse and Konwar^23^. Synthesized particles were checked by dynamic light scattering (DLS) and transmission electron microscope (TEM). The detailed synthesis methods and characterization results are presented in the Electronic Supplementary Information (ESI).

#### Conjugation of SARS-CoV-2 anti-S1 mAbs, anti-S1 recombinant Nbs, and ACE2 with colloidal gold nanoparticles

ACE2 was passively adsorbed to AuNPs (we tried covalent conjugation for ACE2 but passive absorption showed better test results when applied to the test strips), while Nbs and mAbs were covalently conjugated to AuNPs. Before passive conjugation of ACE2 to gold nanoparticles, optimization conditions (pH and protein concentration) were adjusted by using a salts aggregation test. Different concentrations of ACE2 protein (10, 20, 30, 40, and 50 µg/ml) were tested with serial pH conditions (5.5 till 10) of AuNPs. 10 µl of a 10% NaCl solution was added following 30 min incubation at room temperature (RT). The best pH value as well as the optimum concentration of protein for conjugation with colloidal gold was chosen based on the visual absence of aggregation after salts addition. Following the adjustment of the conditions, conjugation by passive adsorption procedures was done according to Tanaka et al.^24^. Briefly, 200 µl of 50 μg/ml of ACE2 has added to 1.8 ml of AuNPs solution (pH 9) and mixed immediately. The mixtures were kept for 2 h at RT with vigorous shaking, then, 200 μl of 10% (w/v) BSA (in 50 mM KH_2_PO_4_ solution pH 9.0) were added to block the non-coated AuNPs surface followed by centrifugation (5000 rpm for 15 min at 4 °C). The pellet containing gold-bio conjugates was then re-suspended using 2 ml of preserving solution (1% (w/v) BSA, 0.05% and 20 mM Tris–HCl buffer, pH 8.2) and kept at 4 °C in dark bottles till used. Covalent conjugation of AuNPs with Nbs or mAbs was carried out using the gold conjugation kit (ab154873, Abcam-UK) according to the manufacturer’s protocol. The produced gold-bio conjugates were checked by Ultraviolet and visible absorption spectroscopy (UV–Vis spectroscopy) as represented in ESI.

#### Dot-blot assay for testing the reactivity of the conjugated probes

The dot-blot assay was performed to test whether the Nbs, mAbs, and ACE2 retain their functionality (antigen binding) after conjugation to AuNPs or not. Moreover, the assay would evaluate the intensity of the reaction between the S1 antigen and subjected proteins before and after conjugation with gold nanoparticles. Dot-blot assay was performed according to Gossen et al.^25^ with some modifications. Briefly, 1 µg of SARS-CoV-S1 antigen was spotted onto a nitrocellulose membrane (NC) and dried for 30 min at RT. Membranes were blocked for 90 min with 2 ml of 3% skimmed milk. 200 µl of previously optimized concentrations (50, 30, and 50 µg/ml) for Nbs, mAbs, and ACE2 respectively were incubated for 30 min with 40 nm AuNPs in a final volume of 2 ml. 10 µl/spot of each conjugated protein solutions were applied on the membranes and left to dry for 2 h at RT, then membranes washed by PBS (0.01 M, pH 7.4) containing sodium dodecyl sulfate (0.1w/v) and kept overnight at RT. As a positive control, the free Nbs & mAbs or ACE2 were incubated as well on the antigen blot. Next day, 10 µl of anti-human IgG-HRP conjugate (1:1000) for ACE2, anti-rabbit IgG-HRP (1:20,000) for mAbs, and anti-llama IgG-HRP (1:10,000) for Nbs were spotted and incubated for 1 h at RT. The signal was developed using the Pierce DAB substrate kit. As a negative control, 1% BSA adsorbed particles were applied while naked AuNPs served as background controls.

#### Preparation of the signal-enhanced lateral flow test (LFT) strip

##### Principle

This sandwich LFT was assembled for the detection of SARS-CoV-2 S1 antigen in saliva samples. AuNPs-Nbs or AuNPs-mAbs were used as detector probes on the conjugate pad while AuNPs-ACE2 was used as a capture probe on the test line. If the sample contains SARS-CoV-2 S1 antigen, it will be detected by AuNPs-Nbs or AuNPs-mAbs on the conjugate pad then the complex will migrate to the membrane-bound AuNPs-ACE2 at the test line to form the signal-enhanced immune-complex. The unbound conjugates will continue to flow forward, binding to the anti-llama antibody on the control line and turning it red. A colored control line is the validity mark for the developed strip however the detection results were negative or positive.

##### Fabrication and optimization

**Figure 1:**
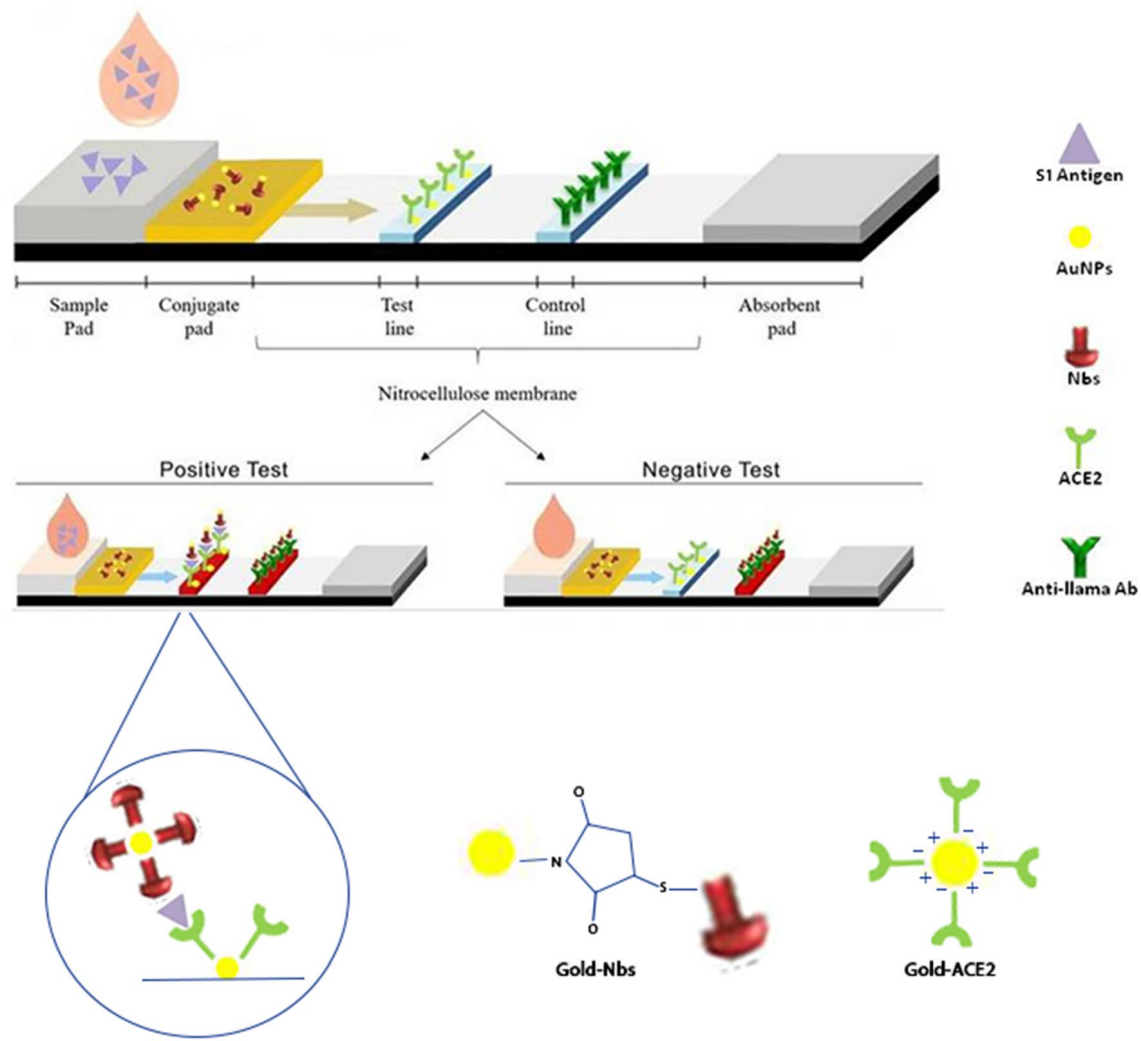
Diagrammatic representation of the Enhanced sandwich LFTS principle for detection of SARS-CoV-2 S1 antigen in saliva samples.

The structure of the developed signal-enhanced LFT strip is presented in Fig. 1. It is composed of four parts, a sample pad, a conjugate pad, a high-flow NC, and an absorption pad. Following several optimization steps, test strips were assembled by dispensing the test line and control line components on the NC membrane. The interval between the two lines was 4 mm. After that, the NC membrane was blocked by a blocking buffer and dried at RT for 1 h. AuNPs-Nbs or AuNPs-mAbs were sprayed on the treated conjugate pad and left to dry for 1 h at 37 °C. The conjugate pad was pasted by overlapping 2.4 mm with the NC membrane, while the sample pad overlapped 3 mm with the conjugate pad. The absorbent pad was pasted on the other side of the NC. The whole assembled strip was 4 × 70 mm in dimension. The prepared test strips were stored in a sealed bag at RT until use.

##### For optimization conditions, we tested the following factors

- *Conjugate pad:* The conjugate pad was treated with 0.1% Triton X-100, and left to dry at RT. Different volumes of AuNps-Nbs or AuNPs-mAbs (5, 6,…10 μl/strip) in a stabilizing buffer (20% sucrose solution previously diluted with 50 mM potassium dihydrogen phosphate buffer (pH 7.5) and 20 μl of 2-propanol, were prepared. Each concentration was sprayed on a pretreated conjugate pad, followed by incubation for 1 h at 37 °C in a vacuum oven.
- *Test & control line proteins:* Different volumes (3, 5, 10 μl/strip) of ACE2-AuNPs (test line) as well as anti-llama IgG (3 and 5 μl of 1 and 2 mg/ml) per strip were tested for the control line. Optimum concentrations where a distinct red color of control and test line were selected, following testing the same samples under the same conditions.
- *NC membrane blocking:* Different concentrations of skimmed milk blocking solution (1, 0.5, and 0.3%) and different immersion times (10, 15, and 20 min), were tested.
- *Sample volume and preparation:* Different volumes of saliva samples, applied as such, or diluted in sample buffer (250 mM NaCl, 10 mM Tris HCl, and 200 µg/ml BSA), were tried to determine the optimum conditions for maximum LFT reactivity.

#### LFTS surface characterization

Surface characterization of the test strip to ensure the establishment of conjugated probes on the NC membrane was performed by using SEM and AFM^26^. JEOL JSM5200 –SEM was used to characterize the test strip NC and conjugate pad before and after loading of conjugated probes as well as the test line before and after sample loading in the presence of S1 antigen. AFM was carried out by AFM instrument model of 5600Ls manufactured by Agilent Technology, USA. The analyses were performed in tapping mode in different sizes, using phase contrast and height modes. At least six images of different areas were obtained and the best representative images were selected. The images were processed with Agilent’s PicoView 1.5 imaging and analysis software package.

#### Determination of sensitivity, specificity, and stability testing of the developed strips

The limit of detection (LoD) of γ-radiated SARS-CoV-2 was measured using our developed strips to determine their sensitivity. LoD of viruses was obtained by using serial dilutions of γ-radiated SARS-CoV-2 (2.7 × 10^5^ copies/ml) (hcov-19/Egypt/NRC-03/2020-SARS-CoV2 strain (GIS) AID accession#EPI-ISI-430819). This strain was kindly donated by the Centre of Scientific Excellence for Influenza Virus, Environmental Research Division, National Research Centre-Egypt. The specificity of LFT strips was confirmed by testing for their reactivity against two different corona-related spike antigens (SARS-CoV S1, and MERS-CoV S1), that were prepared in the sample buffer. The developed test strips were tested for their storage stability under different temperatures (at 4 °C, RT, and 37 °C) and periodic times (1 and 2 months). Three positive and three negative control cases were used and tested by stored strips, and the test line intensities were recorded and plotted against the time and the storage condition.

#### Application of clinical samples on the developed strips

Saliva samples from 320 confirmed RT-PCR cases, 180 positive cases (with different Ct values), and 140 negative cases, were tested by the developed strips (Fig. S5). Color intensity (as a volume × 10^5^) of the produced test line was read by a gel documentation system (Gel Doc XR+) (Biorad, USA), using Image lab software.

### Statistical analysis

The enhanced-strip technique was evaluated using RT-PCR as a reference test based on the following accuracy measures: sensitivity, specificity, positive predictive value, negative predictive value, and Cohen’s kappa statistic (κ). The Kappa value was estimated to determine the agreement level between the PCR test and the studied technique. The level of agreement was determined according to the following scale (Landis and Koch^27^).

**Table:** 

k-value	Agreement level
0	Poor
0.01–0.2	Slight
0.21–0.4	Fair
0.41–0.60	Moderate
0.61–0.80	Substantial
0.81–1	Almost perfect

Statistical analyses were performed using the analytical software package (IBM-SPSS) version 23. The receiver operating characteristic curve (ROC) was built to test the characteristics of the enhanced-strip assay. For the stability test, Post hoc least significant difference (LSD) test was used to illustrate the statistical differences among the different time intervals. The regression analysis was applied to estimate the relationship between the intensity and time at different temperatures. Pearson’s correlation coefficient (r) was utilized to correlate the time with the measured intensity.

## Results and discussion

### Preparation and characterization of AuNPs

**Figure 2:**
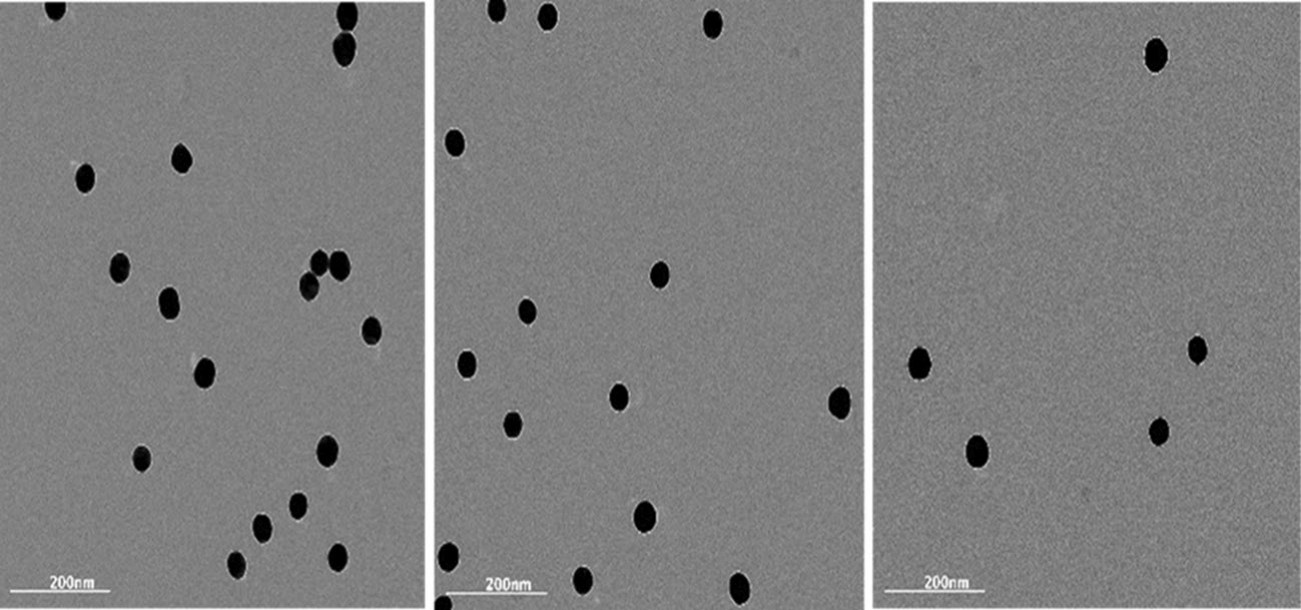
TEM image of spherical shape 40 nm ± 4 nm colloidal gold nanoparticles.

TME image of AuNPs (Fig. 2) showed a good uniformity and dispersion of the prepared particles with an average size of 40 nm, while zeta potential results (Fig. S1) confirmed strongly anionic stable particles which are suitable for conjugation with proteins.

### Conjugation of AuNPs with Nbs, mAbs, and ACE2 proteins

According to Oliver^28^, efficient labeling of AuNPs with various proteins requires optimized conditions including pH and protein concentration^28^. Optimum conditions for ACE2 conjugation with AuNPs by passive adsorption were obtained by using a concentration of 50 µg/ml, at pH 9 in RT, where no salt aggregation was obtained. Efficient covalent conjugation was performed for mAbs and Nbs with gold nanoparticles. The prepared conjugates were checked by UV–vis spectrophotometer that shows slight shifting in the absorption spectra (525–532 nm) on testing gold conjugated proteins compared with free ones as shown in SEI (Figs. S2–S4).

### Dot blot assay

**Figure 3:**
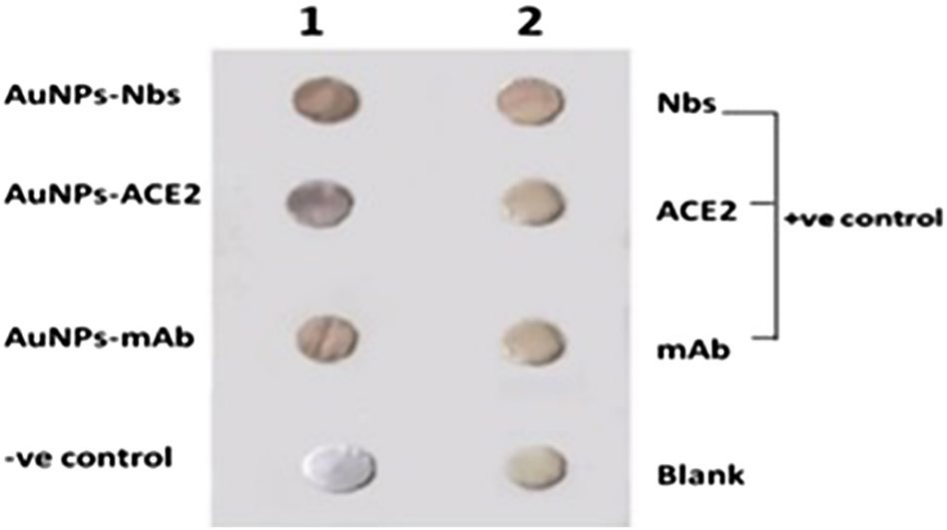
Dot blot assay. The gold-conjugated dots (first three dots in raw 1) represent the gold-conjugated Nbs, ACE2, and mAbs (dark brown dots) respectively followed by the negative control (BSA). The first three colored dots (light brown) in the raw 2, represent the non-conjugated proteins while the last dot represents the blank (gold nanoparticles alone).

As shown in Fig. 3, and Table 1, all conjugated and non-conjugated proteins produced brown color with different degrees of intensity. Furthermore, darker brown color was noticed with conjugated proteins compared to the corresponding non-conjugated ones. The most potent brown color (the highest intensity) was observed with Nbs-AuNPs, denoting that they had the strongest reactivity against the SARS-CoV-2 antigen.

**Table 1:** Color intensity readings of free and gold-conjugated proteins (Nbs, mAbs, and ACE2) following their reaction with SARS-CoV-2 S1 antigen in dot blot assay.

	Nbs		mAbs		ACE2		Blank	Blank
	Free	Gold conjugated	Free	Gold conjugated	Free	Gold conjugated	Gold	BSA
Color intensity	2.31 × 10^5^	3.64 × 10^5^	2.19 × 10^5^	2.86 × 10^5^	1.82 × 10^5^	2.87 × 10^5^	0.46 × 10^5^	0.27 × 10^5^

### Optimization conditions for the developed strip

**Figure 4:**
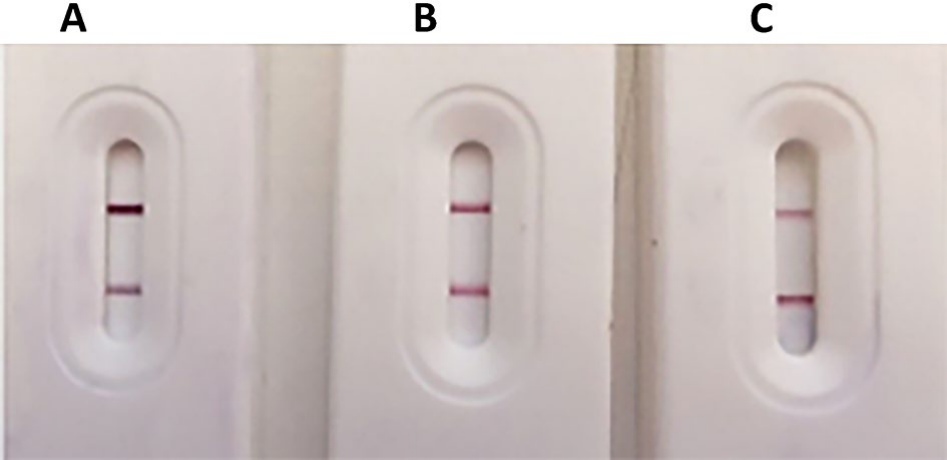
Optimization of the blocking buffer and conditions for an enhanced strip based upon the blocking buffer and the immersion time for the same test sample, (**A**) 50 mM boric acid and 0.3% skim milk and 15 min. immersion time, (**B**) 50 mM boric acid and 0.3% skim milk and 20 min immersion time), (**C**) 50 mM boric acid and 0.3% skim milk and 10 min immersion time. (**A**) Showed the most identified control and test line (strongest red color).

Different optimization conditions were tested to obtain the most efficient and stable version of the developed strip:

1. *Conjugate pad treatment*: The first condition applied was the pre-treatment of the conjugate pad using sucrose stabilizing buffer, which serves as a re-solubilization and a preservative agent. This was performed to maintain the functionality of the detector particles, and the consistency of conjugate release, which are essential for all LFTs^29^.
2. *Optimum concentrations of the reagents applied on the LFT strip:* For conjugate pad reagents, 5 and 8 μl/strip of AuNPs-Nbs or AuNPs-mAbs respectively, were selected, while, 5 μl of AuNPs-ACE2 on the test line, and 1 mg/ml of anti-llama IgG on the control line, were chosen.
3. *The optimum blocking buffer:* for the NC membrane was 0.3% skim milk in 50 mM boric acid buffer (pH 8.5), with strip immersion for 20 min (Fig. 4).
4. *Sampling conditions:* 30 μl of saliva diluted with sample buffer (1:1).

### Morphological characterization of the LFT strip

**Figure 5:**
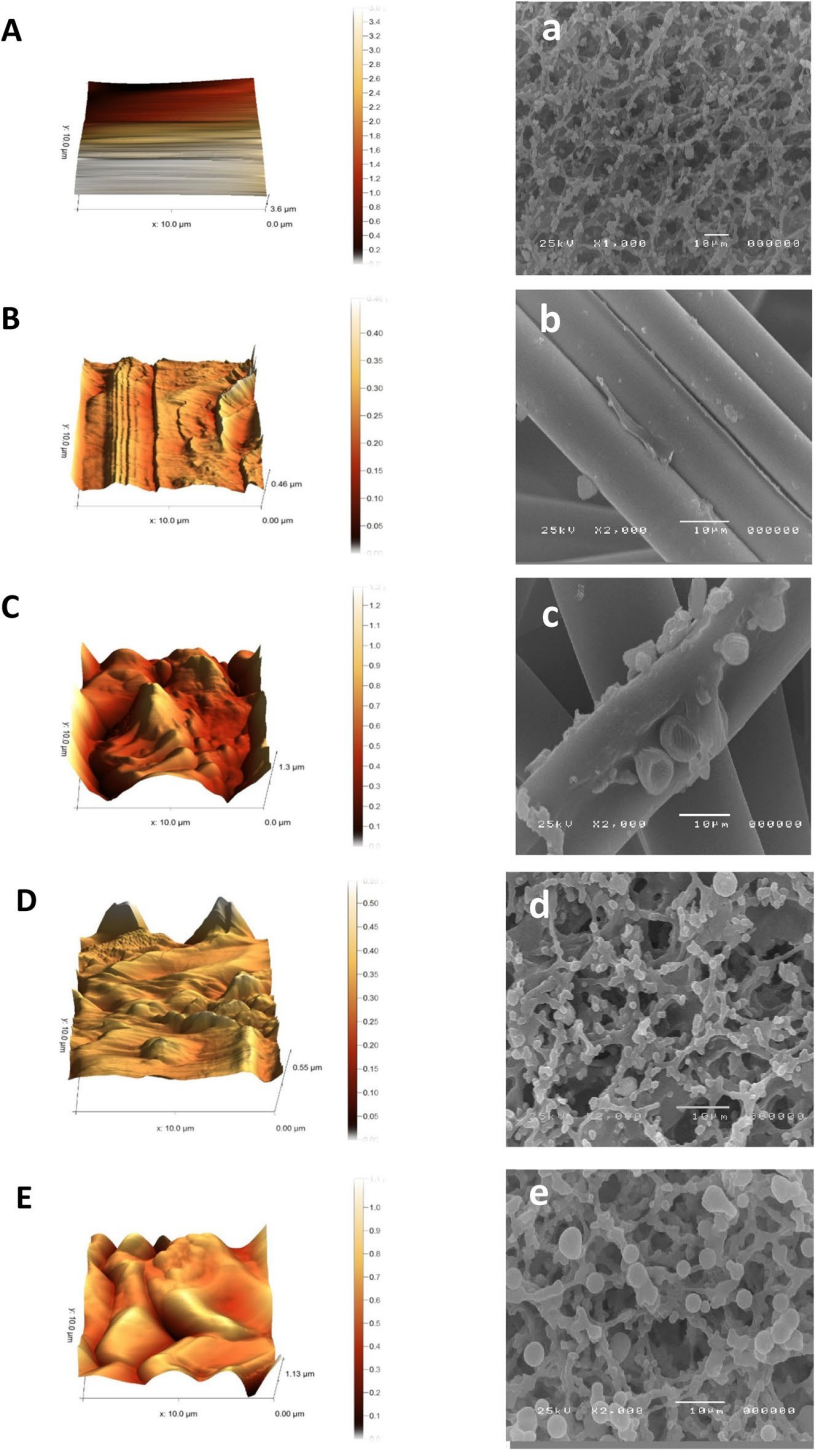
Morphological analysis by AFM (**A**–**D**) and SEM (**a**–**d**) of the membrane surfaces used for LFT construction. NC membrane (**A** and **a**), conjugate pad before (**B** and **b**) and after loading of the detector conjugate (**C** and **c**), test line with capture conjugate (**D** and **d**), test line with sandwich complex (**E** and **e**). Detector conjugate: AuNPs-Nbs on the conjugate pad. Capture conjugate: AuNPs-ACE2 on the test line. Sandwich complex: S1 antigen in positive samples, sandwiched between the detector and capture conjugates on the test line.

AFM and SEM are widely-used techniques for the morphological analysis of different types of surfaces. AFM images, as shown in Fig. 5A–E, representing the morphologies for NC membrane and conjugate pad used in the construction of the LFT strip, before and after loading of gold conjugated proteins (Nbs and ACE2), as well as the modification after sample loading. The smoother surface morphology of bare NC is shown in Fig. 5A. The conjugate pad morphological characterization is shown in Fig. 5B and C before and after the loading of AuNPs-Nbs respectively. The bare conjugate pad showed a structure of 0.46 μm, while an increase in the height was observed following the loading of the gold-conjugated nanobodies with a structure of 1.3 μm. Figure 5D shows the morphology of the AuNPs-ACE2 immobilized at the test point forming globular phases on the NC membrane consisting of structures of 0.55 μm. After contact with the saliva sample containing the S1 antigen, a sandwich complex was formed at the test line that showed a higher density of these smaller globules with a structure of 1.3 μm as shown in Fig. 5E, confirming the immobilization of the detector probe on the conjugate pad surface. The top-view SEM images are shown in Fig. 5a–e. The NC membrane had a uniform porous structure with 8-μm pores approximately (Fig. 5a), this ensured that the antigen-detector probe complex will flow smoothly and had adequate time for interaction with the capture probe. The conjugate pad morphology before and after AuNPs-Nbs loading is represented in Fig. 5b and c, confirming the successful loading of the detector probe. Test line morphology before and after the complex formation with S1 antigen is represented in Fig. 5d and e respectively.

### Determination of sensitivity, and specificity of the developed LFT strip

**Figure 6:**
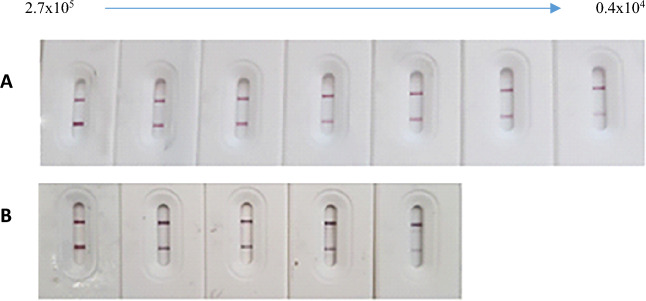
Limit of detection (LoD) for Au-Nbs (**A**) and Au-mAbs strip (**B**), by using serial dilutions of inactivated SARS-CoV-2 starting from 2.7 × 10^5^ copies/ml.

**Figure 7:**
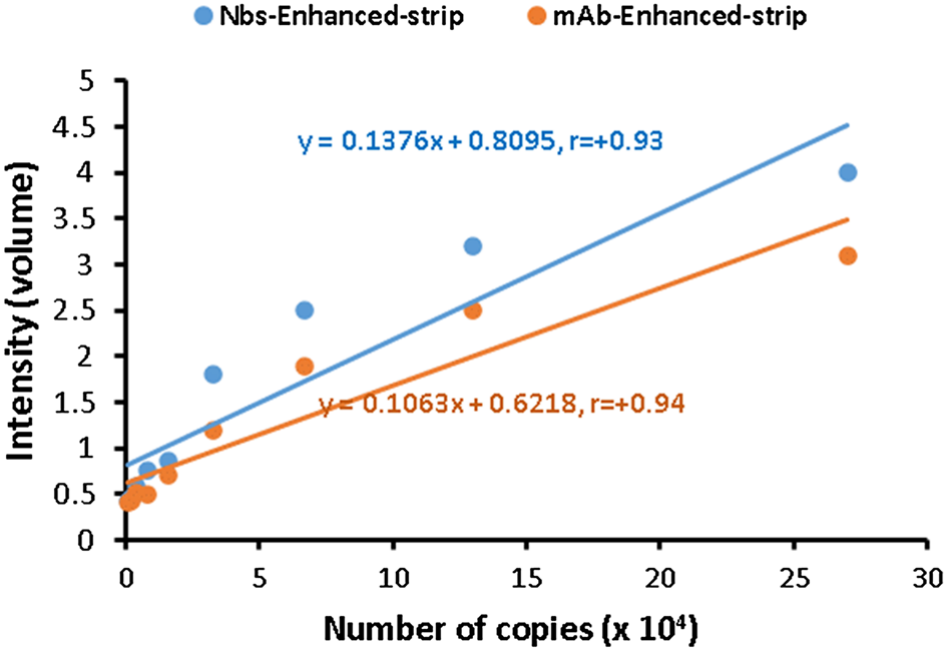
The relationships between the intensity of color in the enhanced LFT strip, using Nbs or mAbs, and the number of viral copies in saliva samples. r: represent Pearson’s correlation coefficient.

The limit of detection (LoD) for virus particles was 0.4 × 10^4^ copies/ml for AuNPs-Nbs strips, and 1.6 × 10^4^ copies/ml for AuNPs-Mabs strips (Fig. 6). Furthermore, the relationships between the intensity and number of viral copies are presented (Fig. 7). Strong positive correlations were observed between the number of viral copies and the intensity readings. Compared to other studies, our results reveal a noticeable improvement in the sensitivity of AuNPs-Nbs strips. Zhang et al.^30^ developed a lateral flow test strip for the detection of COVID-19 proteins (RBD & N) using AIE luminogens (AIEgens) as reporters. They reached 6.9 ng/ml antigen detection concentration for RBD protein and 7.2 ng/ml for N protein^30^. Moreover, Lee et al.^31^, employed ACE2 and anti-S1mAb conjugated with red cellulose nanobeads, as capture and detection probes respectively, in LFT strips. They reported that the LoD of their test was 1.86 × 10^5^ copies/ml^31^. On application of the developed LFT strips for testing the cross-reaction with other rather than SARS-CoV-2 antigens, no cross-reaction with SARS-CoV or MERS-CoV antigens was detected.

### Determination of stability and reproducibility of the developed LFT strip

**Figure 8:**
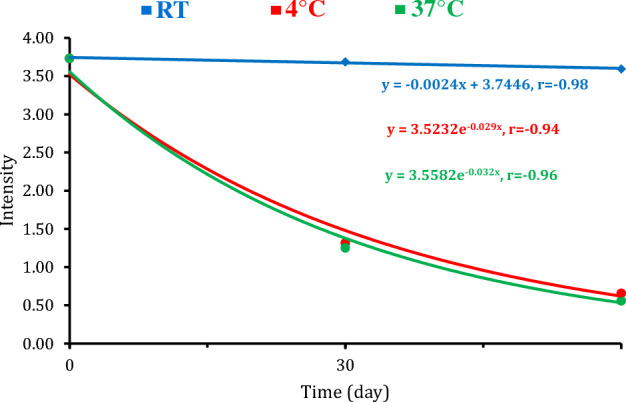
Relationship between color intensity and time, at different storage temperatures.

**Figure 9:**
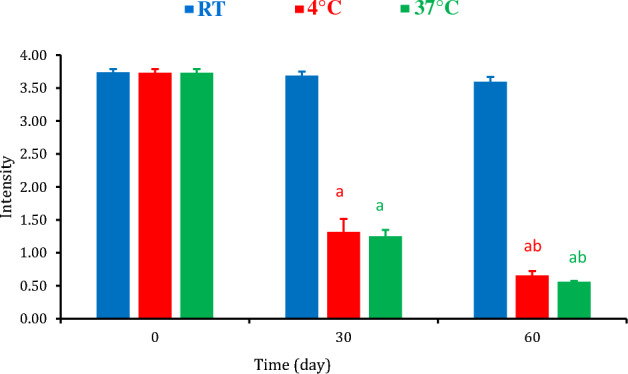
The intensity at different temperatures and different time intervals. Data are displayed as mean ± standard deviation. (**a** and **b**) Represent significant differences (p < 0.05), as compared to the 0 and 30 days, respectively.

Following the testing of three positive cases by test strips after different storage conditions, the regression and correlation analyses of the test line color intensities were inversely correlated with the time and showed strong negative correlations at room temperature (r = − 0.97), at 4 °C (r = − 0.94), and 37 °C (r = − 0.96) (Fig. 8). By the 30th day, color intensity was significantly declined at 4 °C and 37 °C, gradually, and showed the lowest values on the 60th day (Fig. 9). These results revealed that the best stability was obtained for the strips stored at RT, showing great reproducibility for up to two months comparing with one month only for mAbs based strip. For those stored at 37 °C or at 4 °C, the strips showed weak signal intensity after one month (Fig. S6).

### Application of clinical samples

**Figure 10:**
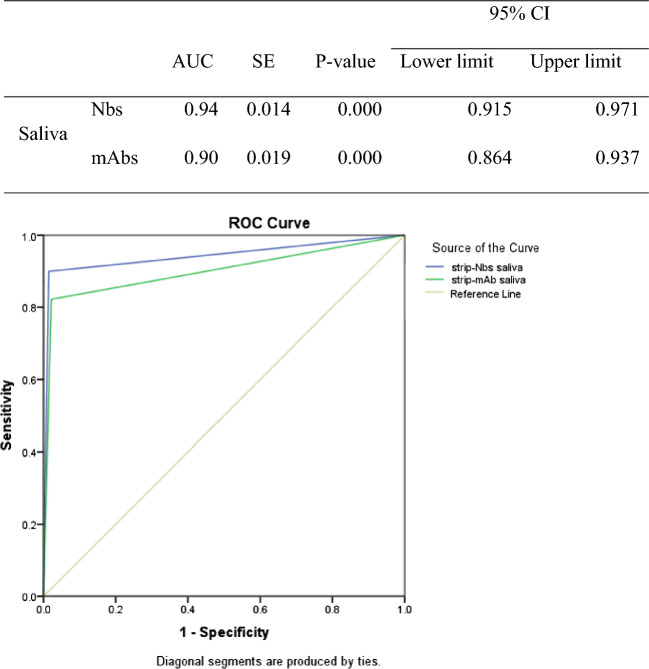
Receiver operating characteristic (ROC) curve of enhanced strip assay using Nbs or mAbs in saliva samples with RT-PCR as a reference test. AUC: area under the curve.

**Figure 11:**
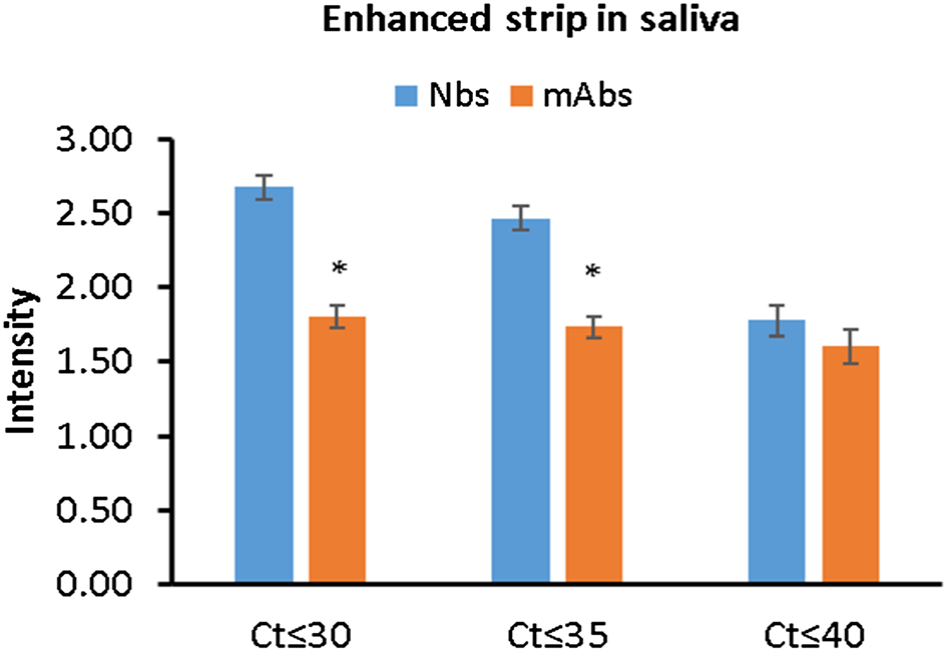
Color intensity (volume × 10^5^) of the Enhanced LFT strip test line for detection of S1 antigen in saliva samples with different Ct values using either nanobodies (Nbs) or monoclonal antibodies (mAbs). Bars represent mean ± standard error. *Represents a significant difference (p < 0.05), as compared to the enhanced strip with Nbs.

The positivity of symptomatic COVID-19 patients was confirmed by RT-PCR, the gold standard test, and their viral load values (Cts) were estimated. The collected saliva samples of these symptomatic cases were tested for the presence of S1 antigen using our developed signal-enhanced strips. ROC curve analysis showed a higher value of the area under the curve (AUC) for the Nbs- based LFT strip than that for mAbs-based strips (Fig. 10). Accuracy measures of Nbs-based strip and mAbs-based strip, using RT-PCR as a reference test, are displayed in Tables 2 and 3. In early detected subjects with Ct values $\le$ 30, Nbs-based strips exhibited higher sensitivity (97.14%) than mAbs-based strips (83.33%). For most Ct values of Nbs- based strips, the kappa statistic was always greater than 0.8, reflecting a perfect agreement level with RT-PCR results. This improvement in sensitivity could be attributed to the advantageous characteristics of nanobodies including their high stability, small size, and marked affinity to their specific antigen^11^. Moreover, the high sensitivity of the developed test strip could be ascribed to the application of the signal-enhanced system. This is in agreement with the work of Tanaka et al.^24^, who claimed that a signal-enhanced strip, containing dual gold conjugated detector and capture probes, guarantees a highly sensitive immunochromatographic assay. The color intensity of the test line in the LFT strip is proportional to the antigen concentration of positive samples. The color of intensity at different Ct values, using Nbs or mAbs, are displayed in Fig. 11. At most Ct values, the Nbs-based strip showed significantly higher color intensity than a mAbs-based strip.

**Table 2:** Accuracy measures of the Nbs-based versus mAbs-based LFT using RT-PCR as a reference test.

			LFT strip (−)/PCR (−)	LFT strip (+)/PCR (+)	Sensitivity (%)	Specificity (%)	PPV (%)	NPV (%)	Accuracy (%)	kappa	Kappa SE	P-value
In saliva	Overall Ct	Nbs	138/140	165/180	91.66	98.57	98.78	88.46	94.75	0.875	0.027	0.000
		mAbs	137/140	150/180	83.33	97.86	98.01	81.07	89.06	0.783	0.034	0.000
	At Ct $\le$ 30	Nbs	138/140	68/70	97.14	98.57	97.01	96.50	96.67	0.924	0.028	0.000
		mAbs	137/140	63/70	90.04	97.86	95.31	93.84	94.29	0.869	0.037	0.000
	At Ct $\le$ 35	Nbs	138/140	60/65	92.31	98.57	96.77	96.50	96.59	0.92	0.03	0.000
		mAbs	137/140	55/65	84.62	97.86	94.83	93.20	93.66	0.849	0.04	0.000
	At Ct $\le$ 40	Nbs	138/140	37/45	82.22	98.57	94.87	94.52	94.59	0.846	0.047	0.000
		mAbs	137/140	32/45	71.11	97.86	91.43	91.3	91.35	0.746	0.06	0.000

Positive cases about Cycle threshold (Ct).

**Table 3:** Sensitivity and specificity of LFT strips at different Ct values using either Nb or mAb.

	Sensitivity			Specificity		
	Ct ≤ 30 (%)	Ct ≤ 35 (%)	Ct ≤ 40 (%)	Ct ≤ 30 (%)	Ct ≤ 35 (%)	Ct ≤ 40 (%)
Nb-LFT strip	97.14	92.31	82.22	98.57	98.57	98.57
mAb-LFT strip	90.04	84.62	71.11	97.86	97.86	97.86

## Conclusion

During the COVID-19 pandemic, early diagnosis was extremely critical for containing disease transmission. RT-PCR is the gold standard tool for the diagnosis of SARS-CoV-2 but with diverse limitations. We developed sensitive and rapid signal-enhanced test strips, using dual gold conjugates, for exaggeration of signal intensity. This designed dual-responsive immunosensor comprises a gold-conjugated capture probe (ACE2) as well as a gold-conjugated detector probe (using Nbs or mAbs). Using gold—Nbs conjugate for S1 antigen detection was found to exhibit better sensitivity and specificity characteristics than the gold-mAb conjugate. To the best of our knowledge, we are the first to develop nanobodies-based test strips for rapid detection of SARS-CoV-2 antigens. Furthermore, the use of painless, easily collected saliva samples as a reliable specimen for SARS-CoV-2 S1 antigen detection, will make it possible to quickly and accurately identify cases in our communities. Local development of such efficient, sensitive, and broadly accessible diagnostic tools for rapid diagnosis of SARS-CoV-2 antigen in the Egyptian population, is essential and mandatory for consistent control of this pandemic. Further modification of this signal-enhanced LFTS is our prospect, to better our results by gaining a higher sensitivity as well as specificity for various variants of concern of the virus, to face the steady SARS-CoV-2 variants development.

## Supplementary Information

Supplementary Information.

## Data Availability

All data generated or analyzed during this study are included in this manuscript and its supplementary information file.
