# Supplementary material for: Gold conjugated nanobodies in a signal-enhanced lateral flow test strip for rapid detection of SARS-CoV-2 S1 antigen in saliva samples
Source: Sci Rep. 2023 Jun 30;13:10643. doi: 10.1038/s41598-023-37347-y (PMC10313708; doi:10.1038/s41598-023-37347-y)
Supplement: Supplementary file 1 — Supplementary Information. [file 41598_2023_37347_MOESM1_ESM.docx]

**Electronic supplementary Information**

**Gold Conjugated Nanobodies in a Signal-Enhanced Lateral Flow Test Strip for Rapid Detection of SARS-CoV-2 S1 Antigen in Saliva Samples**

Sara Maher^1^, Manal Kamel^2^, Zeinab Demerdash^3^, Hanan ElBaz ^4^, Omar Sayyouh ^5^, Amany Saad^6^, Noha Ali^7^ , Faten Salah^8^, Shimaa Atta^9^.

1. *Ph.D, Lecturer of Immunology, Immunology Department, Theodor Bilharz Research Institute, Giza, Egypt.* [*Saram6756@gmail.com*](mailto:Saram6756@gmail.com)
2. *M.D, Professor of Immunology, Immunology Department, Theodor Bilharz Research Institute, Giza, Egypt.* [*Baher_ronny@hotmail.com*](mailto:Baher_ronny@hotmail.com)
3. *M.D, Professor of Immunology, Immunology Department, Theodor Bilharz Research Institute, Giza, Egypt.* [*zeinabdem@hotmail.com*](mailto:zeinabdem@hotmail.com)
4. *M.D, Professor of Immunology, Immunology Department, Theodor Bilharz Research Institute, Giza, Egypt.* [*helbaz77@gmail.com*](mailto:helbaz77@gmail.com)
5. *MD, Infection control and clinical Microbiology, Theodor Bilharz Research Institute, Giza, Egypt.* [*o.sayyouh@tbri.gov.eg*](mailto:o.sayyouh@tbri.gov.eg)
6. *M.B.B.CH, Immunology Department, Theodor Bilharz Research Institute,* [*zaheramani0@gmail.com*](mailto:zaheramani0@gmail.com)*.*
7. *M.D. Assistant Researcher of Immunology, Immunology Department, Theodor Bilharz Research Institute, Giza,Egypt* [*dr.nohaali82@gmail.com*](mailto:dr.nohaali82@gmail.com)
8. *PhD, Professor of Immunology, Immunology Department, Theodor Bilharz Research Institute, Giza, Egypt.* [*fatensdm215@gmail.com*](mailto:fatensdm215@gmail.com)
9. *M.D, Associate Professor of Immunology, Immunology department, Theodor Bilharz Research Institute, Giza, Egypt.*[*attashimaa@yahoo.com*](mailto:attashimaa@yahoo.com)

**Contents**

Synthesis of colloidal gold nanoparticles …………………………………...S1

Results and discussion……………………………………………………….S2

Figure S1. Zeta potential distribution of AuNps measured by Zitasizer…….S3

Figure S2 UV-Vis absorption spectra of ACE2 conjugated AuNPs…………S4

Figure S3: UV-Vis absorption spectra of S1mAbs conjugated AuNPs……...S5

Figure S4: UV-Vis absorption spectra of nanobodies conjugated AuNPs......S6

Figure S5: Application of clinical samples…………………………………. S8

Figure S6: Stability testing for prepared enhanced strip……………………..S9

**Synthesis of colloidal gold nanoparticles (40nm)**

The average particle size of 40 nm colloidal gold nanoparticles was prepared successfully by the chemical reduction method. The protocol was adopted from Frens et al (1973) with slight modifications according to Borse and Konwar., 2020. Briefly, aqueous stock solutions (2%) of HAuCl4 and (1%) trisodium citrate (TSC) were prepared. 98 ml of distilled water (DW) and 2 ml of 1% HAuCl4 were added to a 500 ml conical flask. The solution was mixed thoroughly and refluxed with constant stirring. 3 ml of 1% TSC was added immediately to the conical flask with continuous stirring. Within 2–5 min, the initial pale yellow-colored solution turned colorless and changed to bluish-gray. Five minutes later, the solution turned reddish-purple indicating the formation of AuNPs. The solution was stirred for another 10 min and cooled to room temperature. Prepared particles were characterized using DLS&TEM.

**Results**


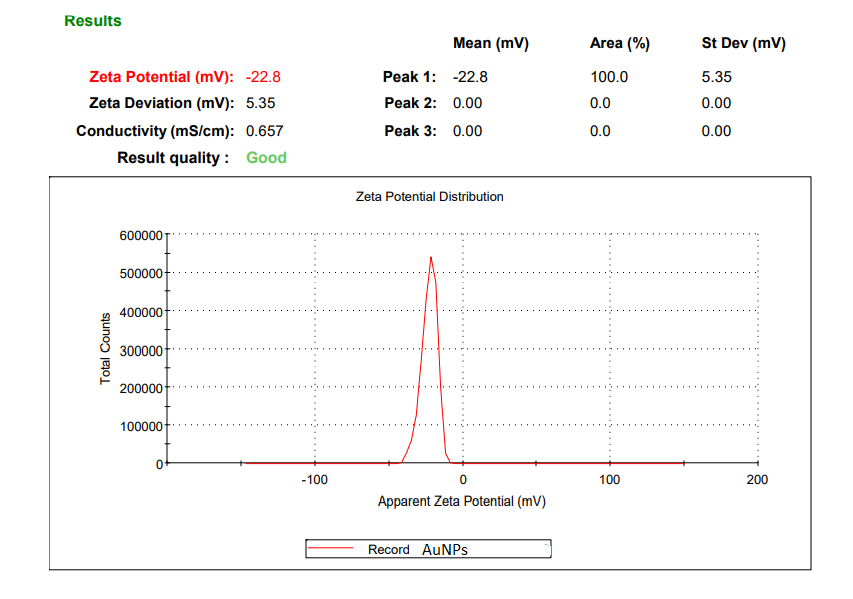


**Figure S1:** Zeta potential distribution of AuNps measured by Zitasizer

**
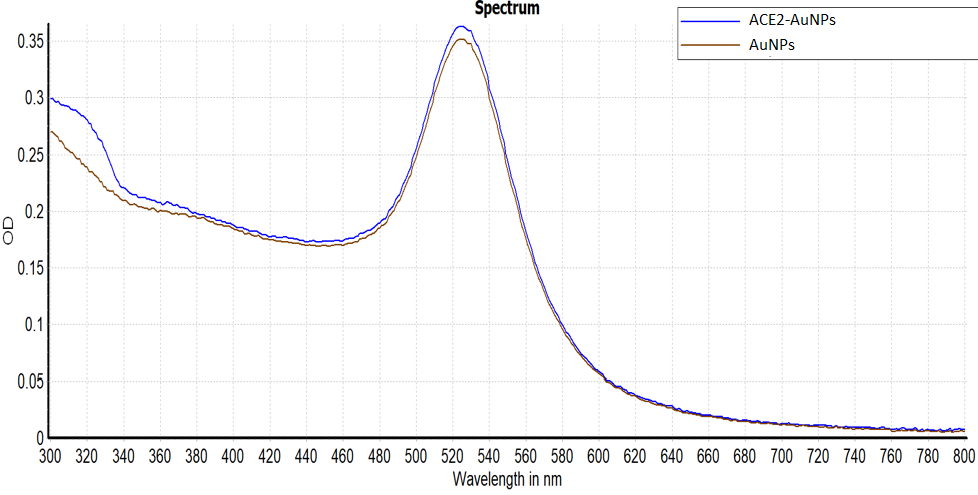
**

**Figure S2:** UV-Vis absorption spectra of ACE2 conjugated AuNPs


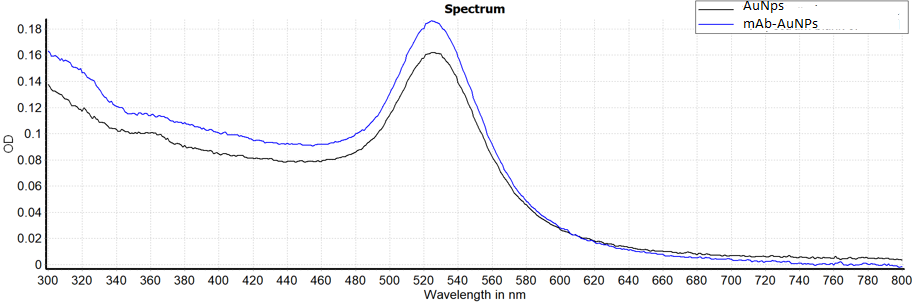


**Figure S3:** UV-Vis absorption spectra of S1mAbs conjugated AuNPs (mAbs-AuNPs)

**Figure S4:** UV-Vis absorption spectra of nanobodies conjugated gold nanoparticles (Nbs-AuNPs )

**Figure S5:** Application of some confirmed RT-PCR positive and negative saliva samples on the developed enhanced strip Nbs or mAbs. For the same group of samples, variable color intensities of test line using (A) Enhanced-Nbs strip (B) Enhanced –mAbs strip and negative samples (C), was displayed with remarkable potent intensity on using Nbs based strip.


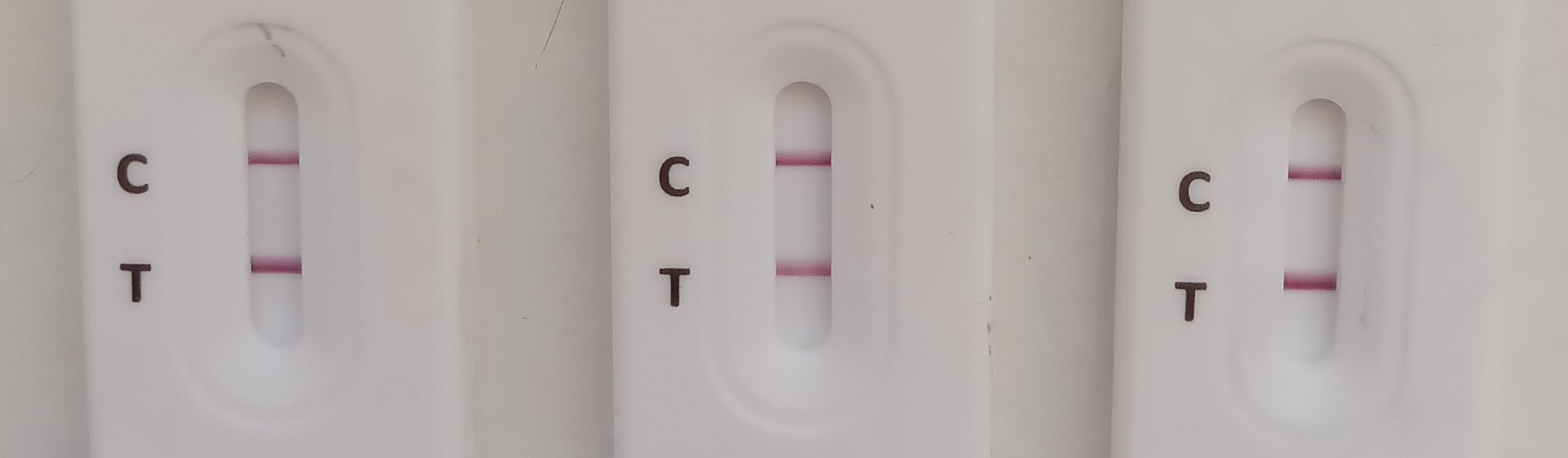

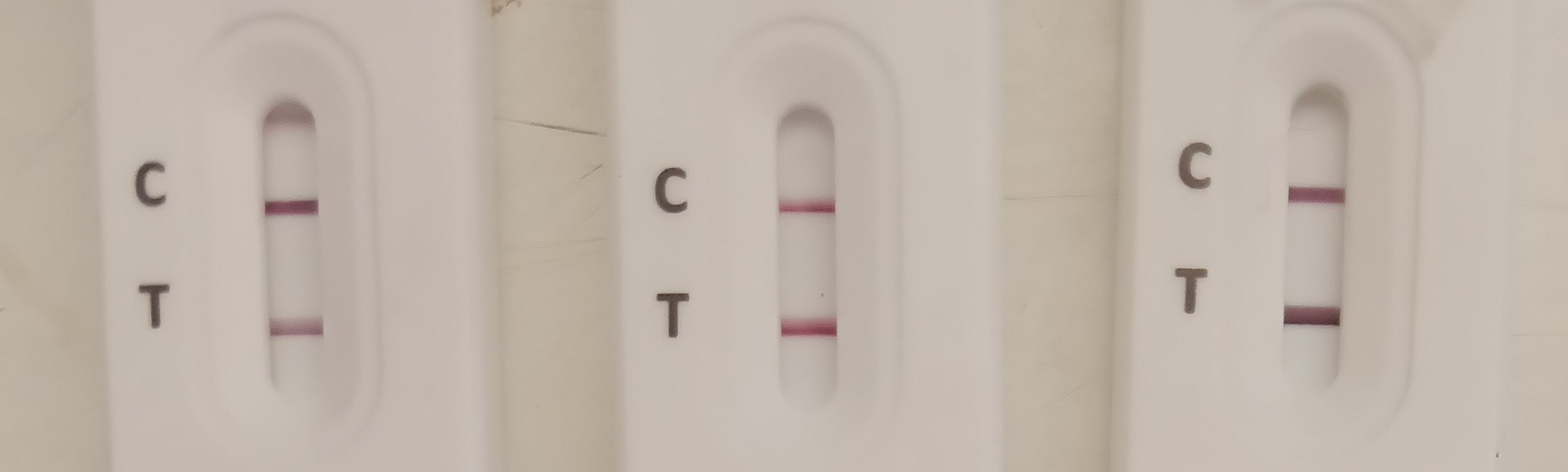


**B**

**A**


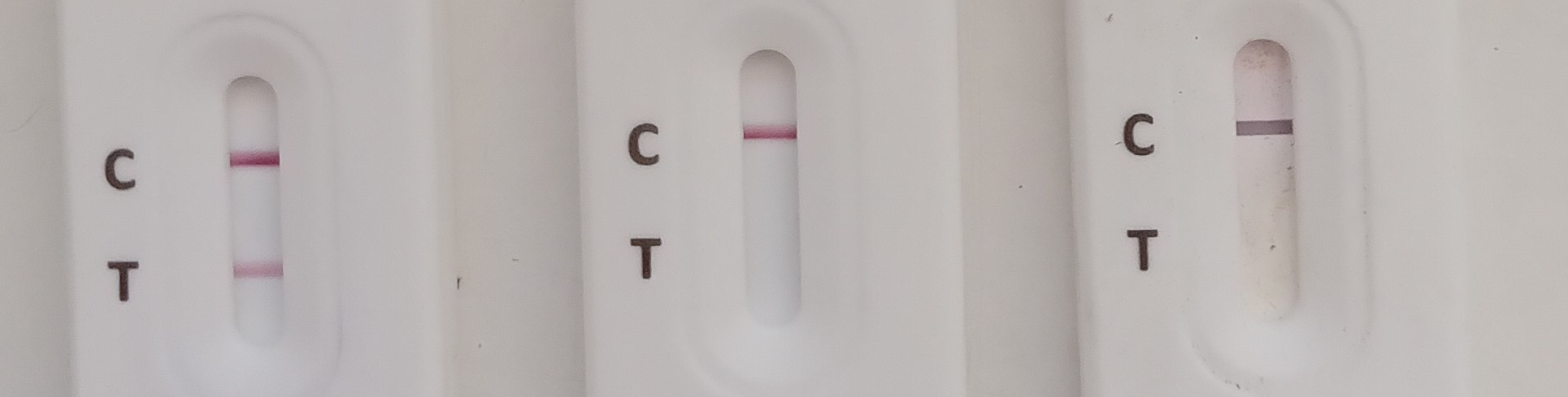


**C**

**D**

**Figure S6:** Stability testing for three samples using Enhanced-Nbs strip under different conditions. **A:** before storage, **B:** storing for two months at RT, **C:** storing for one month at 4^ₒ^C, D: storing for one month at 37C. NB: Best storage conditions was observed after for strips stored at RT (B).
